# Proliferative Verrucous Leukoplakia Presenting as a Ring Around the Collar and Cancer: A Case Report

**DOI:** 10.7759/cureus.56077

**Published:** 2024-03-13

**Authors:** Valentin Vergier, Katarzyna Czarny, Jean-Jacques Brau, François Le Pelletier, Ihsène Taihi

**Affiliations:** 1 Department of Dentistry, Faculty of Health, Université Paris Cité, Montrouge, FRA; 2 Department of Dentistry, Charles Foix Hospital, Assistance Publique-Hôpitaux de Paris, Ivry-sur-Seine, FRA; 3 Orofacial Pathologies, Imaging, and Biotherapies Laboratory (URP 2496 BRIO), Université Paris Cité, Montrouge, FRA; 4 Department of Maxillo-facial Plastic Surgery and Stomatology, Gonesse Hospital, Gonesse, FRA; 5 Odontology and Maxillofacial Prosthesis Unit, Department of Cervicofacial Cancerology, Gustave Roussy Cancer Center, Villejuif, FRA; 6 Department of Pathology, Pitié-Salpetrière Hospital, Assistance Publique-Hôpitaux de Paris, Paris, FRA; 7 Department of Oral Surgery, Rothschild Hospital, Assistance Publique-Hôpitaux de Paris, Paris, FRA

**Keywords:** oral dermatology, attached gingiva, verrucous leukoplakia, oral cancers, squamous cell carcinoma

## Abstract

Proliferative verrucous leukoplakia (PVL) is an oral mucosa lesion with a high rate of malignant transformation. The diagnosis is often difficult, especially when the initial lesion is a simple homogeneous white leukoplakia, and when located only on the gingiva or palate. Moreover, the anatomopathological analysis is non-specific in the initial stages. The gingival PVL localisation (gPVL) is described as the most aggressive form with the highest rate of malignant transformation. Cases with a unique gingival localisation are rare, described with a “ring around the collar” clinical form. Considering the difficulty of early diagnosis of gPVL, we report the case of a 72-year-old woman, who presented “white lesions on her gingiva” with a slight discomfort in February 2019. The lesion was initially limited to the buccal part of the mandibular right gingiva, but rapidly extended to all the lingual and buccal mandibular gingiva during follow-up, leading to a diagnosis of gPVL. Two biopsies were performed, which concluded a verrucous hyperplasia and papilloma with a lichenoid part. The diagnosis of gPVL was made after a six-month follow-up based on clinical and anatomopathological factors. The gPVL transformed into a squamous cell carcinoma (SCC) after 18 months of follow-up. A surgical right mandibular bone excision with an autologous left fibula graft associated with radiotherapy was performed. Three years after the surgery, the patient remains under monitoring, with several gPVL and SCC recurrences treated. This case highlights that gPVL is a rare and aggressive form of PVL, with a high risk of fast malignant transformation. Knowledge about its aetiology, anatomic pathology, and biological markers is highly needed to speed up the diagnosis and develop specific follow-up and treatment.

## Introduction

Proliferative verrucous leukoplakia (PVL) is a white oral mucosa lesion and a potentially malignant disorder. It was first described in 1985 by Hansen et al. [1], who described the lesion as “a simple hyperkeratosis but tends to extend and become multifocal over varying periods of time. The lesions are slow-growing, persistent, irreversible, and frequently developed erythematous components. Some areas later become exophytic and wart-like and transform into lesions that are clinically and microscopically identical to verrucous carcinoma (VC) and squamous cell carcinoma (SCC). In addition, they are resistant to different treatments.”

A five patterns and 10 grades scale simplified later to a four patterns and five grades scale has been proposed [2]. The grades range from normal mucosa (grade 0) to hyperkeratosis (grade 1), verrucous hyperplasia (grade 2), verrucous carcinoma (grade 3), and, lastly, invasive SCC (grade 4).

The diagnosis of PVL is often difficult and needs a close follow-up to control the lesion’s evolution. Indeed, in the few cases described in the literature, the diagnosis was made after several months of follow-up because of the challenging diagnosis of the lesion, especially at early stages. Although several diagnostic criteria have been described, they are often unclear and difficult to assess. The most recent and easy to assess are those from Cerero-Lapiedra et al. in 2010 [3]. They classified the criteria as major or minor. For a diagnosis of PVL, the lesion needs to meet three major criteria (the histology criteria among them) or two major and two minor criteria.

Although the majority of reported oral PVL lesions are often multifocal, gingival-only forms have been recently described [4]. The gingival localisation is one of the most frequent and seems to have a higher malignant transformation rate than other localisations [5]. However, very few cases have been described in the literature, especially when the lesion is limited to the gingiva, which makes the diagnosis more difficult. Based on this observation, we report the case of a woman presenting a gingival-only PVL (gPVL) with a rapid malignant transformation.

This case was previously presented as a poster at the 2021 annual French Society of Oral Surgery Congress on September 10, 2021.

## Case presentation

A 72-year-old woman presented at an oral surgery consultation in February 2019, referred by her dentist for “white lesions on her gingiva” with slight discomfort. No history of tobacco or alcoholic intoxication was reported by the patient.

The patient reported a history of balanced hypertension, hypothyroidism, and osteoporosis treated with alendronate. She was followed by a dermatologist for a facial basal cell carcinoma two years ago, treated by photodynamic therapy at that time. Her father and grandfather had carcinomas.

**Figure 1:**
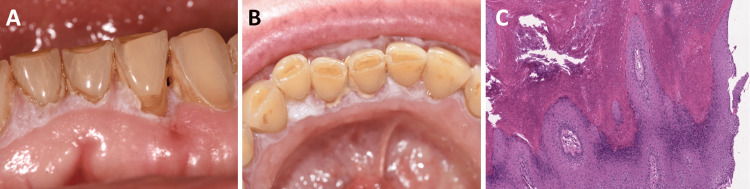
The gingival-only proliferative verrucous leukoplakia case reported before malignant transformation. A: The lesion noted seven days after the first biopsy in February 2019. The specific aspect of a “ring around the collar” leukoplakia can be noticed. B: The lesion noted six months later. It had extended to the lingual mandibular gingiva. C: Hematoxylin-eosin colouration of the second biopsy. The papillomatous aspect with a lichenoid part can be noticed but without any malignant cells.

Clinical examination showed about 1 cm^2^ of gingival linear keratotic plaques with undefined limits and erythematous borders, limited to the attached gingiva, located on the buccal side of the first left mandibular premolar which continued to the first right mandibular molar (Figure 1, Panels A, B). The lesion was attached to the gingiva, and no bleeding on probing, nodules, lymphadenopathy, or ulceration was noted.

At this stage of examination, the possible differential diagnoses were hyperplastic oral lichen planus, PVL, drug-induced leukoplakia, chronic candidiasis, idiopathic leukoplakia, verrucous carcinoma, or SCC.

A biopsy of the gingival papilla was performed in the incisors region. The anatomopathological examination showed papillomatous epithelial hyperplasia and non-specific fibrous reshaping. An idiopathic leukoplakia was diagnosed and a six-month follow-up was established.

During this period, the lesion extended all over the buccal mandibular gingiva and thickened on the lingual side. The lesion grew in thickness at the biopsy site. The patient still did not experience any pain, ulceration, nodules or lymphadenopathy. A second biopsy was performed in March 2020 on the lingual gingival papilla in the incisors region, which concluded verrucous hyperplasia and papilloma with a lichenoid part, without any malignant cells (Figure 1C). The detection of a verrucous part in that biopsy compared to the first biopsy was the major evolution.

Given the histopathological findings and the clinical evolution of the lesion, the gPVL diagnosis was made. At this stage, the patient met four major criteria proposed by Cerero-Lapiedra et al. [3] (Table 1). A leukoplakia lesion with more than two different oral sites (buccal marginal gingiva in incisors and molars regions), the lesions had spread or engrossed during the development of the disease (at the incisors’ lingual marginal gingiva), recurrence in a previously treated area (recurrence after the biopsy), and compatible histopathology with verrucous hyperplasia. The patient also met two minor criteria, i.e., the patient was female and a non-smoker.

**Table 1:** The diagnostic criteria for proliferative verrucous leukoplakia. These criteria were proposed by Cerero-Lapiedra et al. in 2010 [3]. The positive diagnosis is made if the patient meets either three major criteria (histopathological criteria among them) or two major criteria (histopathological criteria among them) and two minor criteria.

Major criteria		Minor criteria	
A	A leukoplakia lesion with more than two different oral sites, which is most frequently found in the gingiva, alveolar processes, and palate	a	An oral leukoplakia lesion that occupies at least 3 cm when adding all the affected areas
B	The existence of a verrucous area	b	A female patient
C	The lesions have spread or engrossed during the development of the disease	c	A non-smoker patient (male or female)
D	There has been a recurrence in a previously treated area	d	A disease evolution higher than 5 years
E	Histopathologically, ranging from simple epithelial hyperkeratosis to verrucous hyperplasia, verrucous carcinoma, or oral squamous cell carcinoma, whether in situ or infiltrating		

The variation of the histological description by the pathologists was due to the similarity of lichenoid lesions and papilloma with PVL at early stages.

Because of the localisation of the lesion as a “ring around the collar” of all the mandibular teeth [4], a total excision could not be performed, despite the high risk of malignant transformation. Thus, a three-month control interval was established to diagnose the malignant transformation as soon as possible.

Between the controls, she was prescribed antibiotics by her generalist due to “stinks in her mouth.” The patient started to experience slight pain but did not report it to her generalist. The clinical examination showed a gingival swelling in the right mandibular premolars and probing of 15 mm but without any signs of general alteration as nodules or facial swelling. This suggested a periodontal abscess. She underwent an ultrasonic scaling in the Periodontology Department in July 2020, along with a seven-day course of amoxicillin and clavulanic acid.

Due to the COVID-19 pandemic, the patient did not come back for three months, although the swelling started to extend to the mandibular molar region. During this period, the patient had a basal cell carcinoma on the nose, which was excised by her dermatologist and analysed.

**Figure 2:**
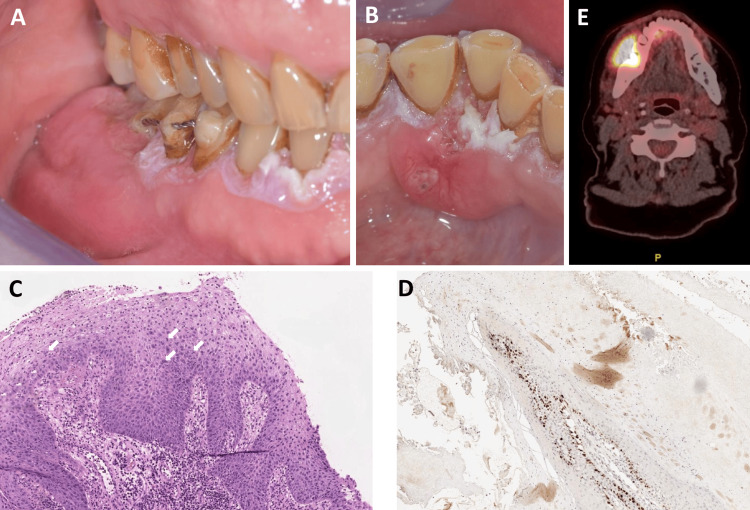
The gingival-only proliferative verrucous leukoplakia case reported during the malignant transformation. A: The lesion in September 2020. Swelling in the vestibular part of the tumefaction in regard to the second right mandibular premolar and second right mandibular molar. B: The lesion in September 2020. Second localization in the lingual gingiva between the second right mandibular incisor and right mandibular canine. C: Biopsies in October 2020. Vestibular biopsy between the second right mandibular premolar and first right mandibular molar: in situ squamous cell carcinoma (SCC) characterised by architectural disorganisation and cytonuclear atypia/mitosis (white arrows) found throughout the epithelium. D: Biopsies in October 2020. Immunohistochemistry DAB anti-Ki67 in the 42-43 site showing hyperplasia with hyperkeratosis but without dysplasia. The site needed a second biopsy to confirm the in situ SCC, which was performed under general anaesthesia at Gustave Roussy Institute. E: Positron emission tomography scan showing the important SCC invasion in the mandibular tissues (Siemens^©^ Biograph Camera, 245 MBq of fludeoxyglucose F18, maximum standardized uptake value = 15.7).

**Figure 3:**
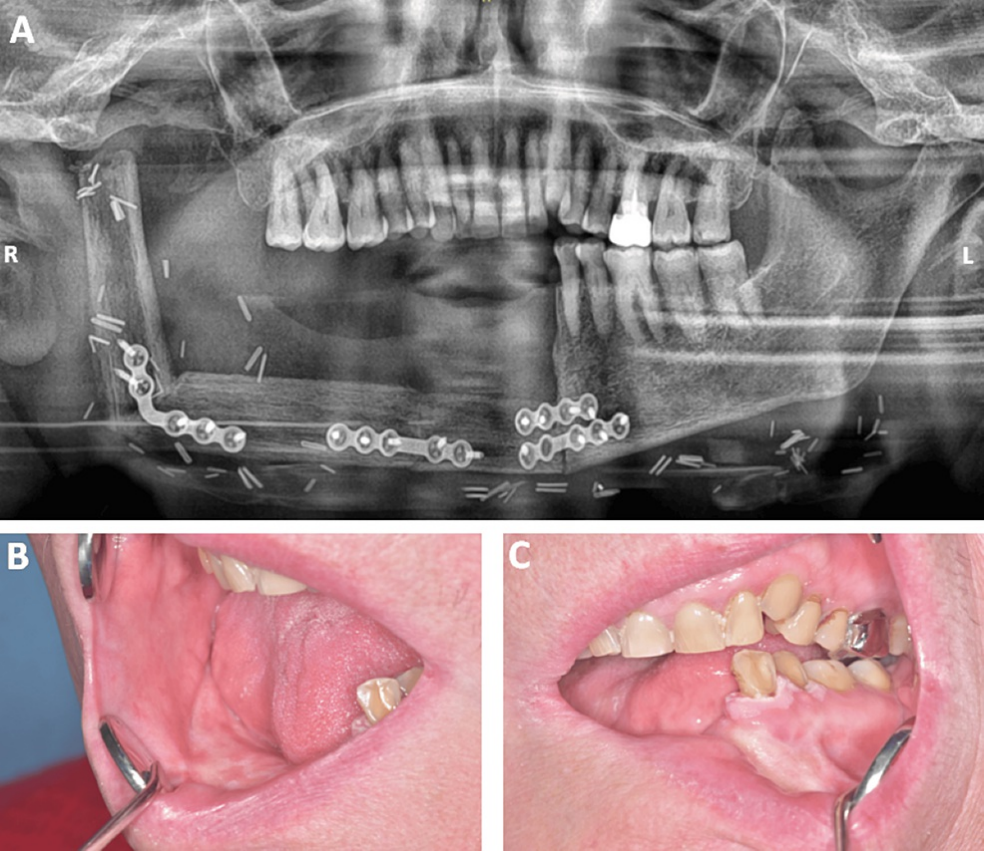
The first malignant transformation treatment. A: Patient’s panoramic radiograph one month after the surgery. B and C: Patient’s intrabuccal view nine months after the surgery. The gingival-only proliferative verrucous leukoplakia persisted even after the surgery and radiotherapy.

The patient came back in October 2020 with a right mandibular facial swelling. Intraoral examination showed suppurative swelling limited to the buccal gingiva regarding the lower right premolars and molars and a second swelling on the lingual side in the lower left incisors (Figures 2A, 2B). This was associated with a right submandibular lymphadenopathy. Given these findings, biopsies were performed and confirmed the diagnosis of SCC (Figures 2C, 2D). The patient was referred to the Cervico-facial Carcinology Department (Gustave Roussy Cancer Center Villejuif, France). After a complete assessment using positron emission tomography scanning (Figure 2E) and local and general radiological analyses, the multidisciplinary consultation staff decided on a surgical right mandibular bone excision with an autologous left fibula graft associated with radiotherapy (Figure 3A). Nine months later, the graft was a success and the patient could drink with her mouth. She continued to eat with gastrostomy for a year and a half after the surgery. She also still had gPVL growing at the left mandibular canine collar, even after the surgery and radiotherapy (Figures 3B, 3C). Two years after surgery, a new SCC was diagnosed on the left mandibular gingiva. The patient was treated for that new lesion with a new autologous right fibula graft, along with radiotherapy. Three years after the first SCC, the patient had a left lymph node recurrence, which was treated with surgery. The patient remains in close monitoring every three months to diagnose every recurrence as soon as possible.

## Discussion

Since gPVL was first described by Hansen et al. in 1985 [1], a few cases have been reported in the literature. This localisation is, however, the most frequent, as 50.9% of PVL cases have at least a gingival localisation [6]. It is important to consider this diagnosis when a verrucous form of marginal linear leukoplakia, evolving rapidly, forming a “ring around the collar” of the teeth appears [4]. It is the first and only case of gPVL we observed in our department, which made the diagnosis even more challenging.

This form of leukoplakia appears mostly in women more than 65 years old, as they represent 67.1% of the PVL cases [6]. Likewise, smoking is not a PVL risk factor, as there is one smoker for two non-smokers in PVL cases [7]. The patient presented here confirms these observations.

Regarding malignant transformation, PVL has a high transformation rate, as 45.8% of PVL cases transform to verrucous carcinoma or SCC [7]. Our patient had a very fast malignant transformation, as it appeared only a year and a half after the first appointment, much faster than the mean follow-up duration of 4.1 years before a malignant transformation, as reported by Palaia et al. [7].

Fortunately, the cancers developed on PVL seem to be less aggressive than other oral SCCs, as only 17.1% of the patients died during follow-up [7-9]. This is also the case of the patient presented here, as she is still alive at the time of this report, three years after the malignant transformation.

The patient presented here also had multiple melanomas before the SCC, in addition to her parents. This could be a sign of inherited genetic factors. To date, there is no evidence of candidate biomarkers which could be predictive of PVL malignant transformation [9]. To our knowledge, the genetic factors have been explored and can be a good area for further studies.

The human papillomavirus (HPV) 16 infection is often evoked as a potential factor for PVL and malignant transformation [10]. As several studies have found no association between viral infection and the appearance of the lesion [4,11], we did not perform an HPV infection analysis for our patient.

The case presented here highlights the difficulty in diagnosing PVL, especially when it is an exclusively gingival localization. Indeed, in the early stages, gPVL is often confused with other oral leukoplakias, such as traumatic or smoking leukoplakias, or drug-induced leukoplakias [2]. The histopathological analysis is also confusing, as it often shows hyperkeratosis or verrucous hyperplasia at the initial stage, as shown in the reported case. Verrucous hyperplasia is characterised by epithelial thickening, which induces a slight lifting of the hyperkeratotic surfaces, with in-depth accentuation of the epithelial basal layer.

Our patient highlights the evolving process of the gPVL and the importance of a close follow-up with regular biopsies in case of extension or change in the clinical presentation.

In our case, the second biopsy diagnosed a papilloma associated with a lichenoid reaction. This was disturbing because of our clinical impression of the gPVL diagnosis. This was recently described in a study that analysed multiple slides labelled as PVL or lichenoid lesions, with 5% of specimens exhibiting both lichenoid lesion and PVL characteristics. This mainly concerned lesions of the same site analysed at different times. This may be due to discrepancies between the diagnoses or changes in the lesion. Consequently, the hypothesis of a continuum between oral lichenoid lesions and PVL, particularly on the same site, should be considered [12]. Indeed, the characteristics of lichenoid lesions are common to the verrucous hyperkeratosis of PVL and verrucous carcinoma; it may, therefore, be a non-specific inflammatory response to dysplasia or malignancy rather than concomitant lichenoid disease [13].

## Conclusions

The diagnosis of gPVL can be quite challenging because of the non-specific histology and clinical aspects. The rapid evolution and the particular form of leukoplakia evolving as a “ring around the collar” on the attached gingiva are key to diagnosing this specific form of PVL. Follow-up and repeated biopsies are very important for PVL, as it can rapidly transform to SCC or verrucous carcinoma with around 50% risk of malignant transformation. Because of very few cases reported in the literature, the knowledge about the aetiology, anatomopathological, or genetic markers of gPVL is very poor. This knowledge can help predict malignant transformation and improve gPVL follow-up to adapt the therapeutic procedures available.

## References

[1] Hansen LS, Olson JA, Silverman S Jr (1985). Proliferative verrucous leukoplakia. A long-term study of thirty patients. Oral Surg Oral Med Oral Pathol.

[2] Czarny K, Le Pelletier F, Vergier V, Taihi I (2022). [Proliferative verrucous leukoplakia]. Ann Dermatol Venereol.

[3] Cerero-Lapiedra R, Baladé-Martínez D, Moreno-López LA, Esparza-Gómez G, Bagán JV (2010). Proliferative verrucous leukoplakia: a proposal for diagnostic criteria. Med Oral Patol Oral Cir Bucal.

[4] Upadhyaya JD, Fitzpatrick SG, Islam MN, Bhattacharyya I, Narayana N, Cohen DM (2021). Marginal linear gingival leukoplakia progressing to "ring around the collar"-an ominous sign of proliferative verrucous leukoplakia. J Periodontol.

[5] Alabdulaaly L, Villa A, Chen T (2022). Characterization of initial/early histologic features of proliferative leukoplakia and correlation with malignant transformation: a multicenter study. Mod Pathol.

[6] Torrejon-Moya A, Jané-Salas E, López-López J (2020). Clinical manifestations of oral proliferative verrucous leukoplakia: a systematic review. J Oral Pathol Med.

[7] Palaia G, Bellisario A, Pampena R, Pippi R, Romeo U (2021). Oral proliferative verrucous leukoplakia: progression to malignancy and clinical implications. Systematic review and meta-analysis. Cancers (Basel).

[8] González-Moles MÁ, Warnakulasuriya S, González-Ruiz I (2020). Clinicopathological and prognostic characteristics of oral squamous cell carcinomas arising in patients with oral lichen planus: a systematic review and a comprehensive meta-analysis. Oral Oncol.

[9] Faustino IS, de Pauli Paglioni M, de Almeida Mariz BA (2023). Prognostic outcomes of oral squamous cell carcinoma derived from proliferative verrucous leukoplakia: a systematic review. Oral Dis.

[10] Palefsky JM, Silverman S Jr, Abdel-Salaam M, Daniels TE, Greenspan JS (1995). Association between proliferative verrucous leukoplakia and infection with human papillomavirus type 16. J Oral Pathol Med.

[11] Fettig A, Pogrel MA, Silverman S Jr, Bramanti TE, Da Costa M, Regezi JA (2000). Proliferative verrucous leukoplakia of the gingiva. Oral Surg Oral Med Oral Pathol Oral Radiol Endod.

[12] Thomson PJ, Goodson ML, Smith DR (2018). Potentially malignant disorders revisited-the lichenoid lesion/proliferative verrucous leukoplakia conundrum. J Oral Pathol Med.

[13] Davidova LA, Fitzpatrick SG, Bhattacharyya I, Cohen DM, Islam MN (2019). Lichenoid characteristics in premalignant verrucous lesions and verrucous carcinoma of the oral cavity. Head Neck Pathol.

